# Supplementary material for: A Novel Genotype of Orientia tsutsugamushi in Human Cases of Scrub Typhus from Southeastern India
Source: Microorganisms. 2025 Feb 4;13(2):333. doi: 10.3390/microorganisms13020333 (PMC11858592; doi:10.3390/microorganisms13020333)
Supplement: Supplementary file 1 [file microorganisms-13-00333-s001.zip › microorganisms-3336791-supplementary.pdf]

Supplementary figure S1

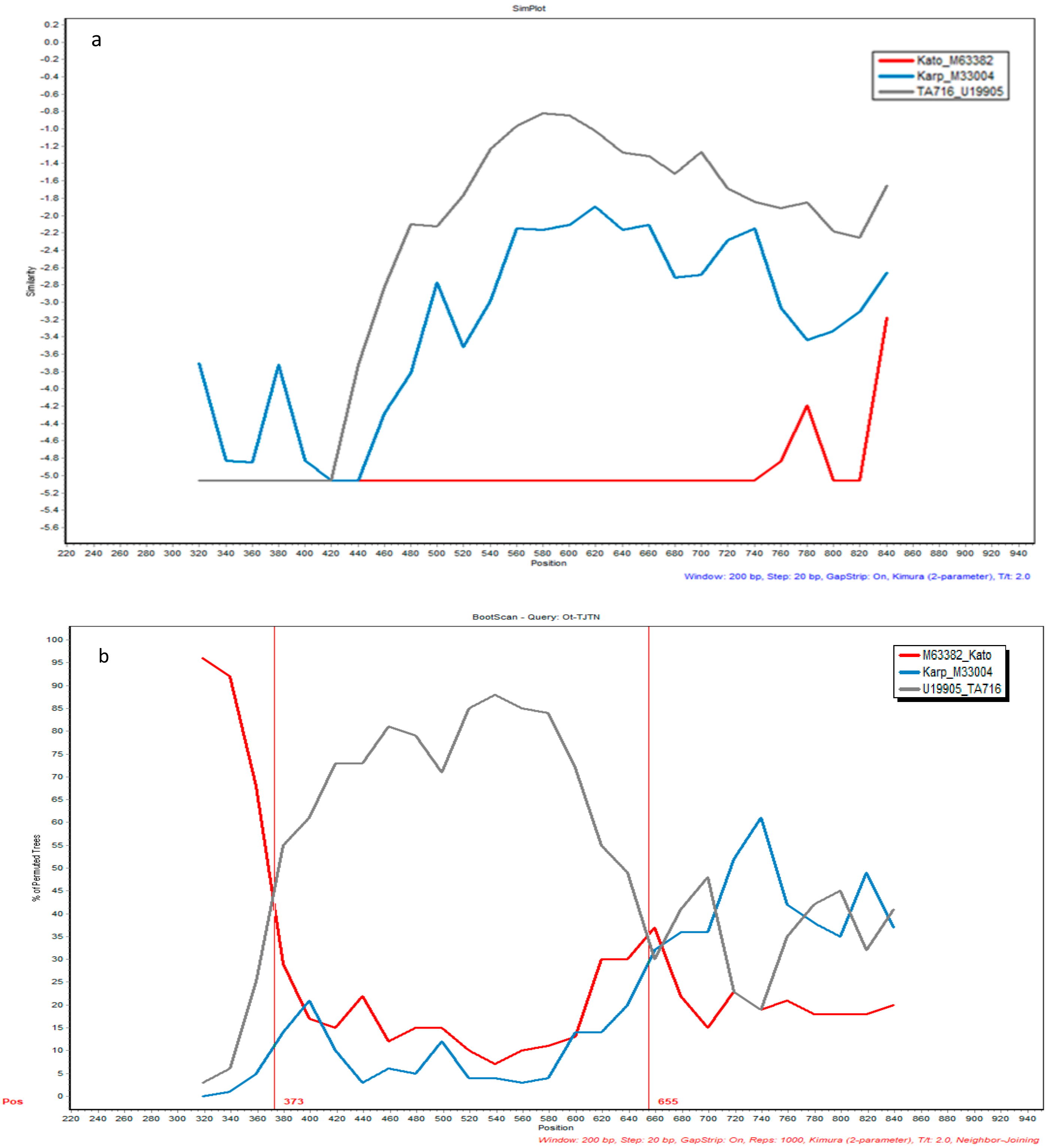

Figure S1. (a) Similarity analysis of Simplot using the new Ot-TJTN TSA 56 kDA sequences as query. TA716, and Kato reference sequences shows an overlapping curve in variable domain-I against the new genotype (Ot-TJTN); (b). SimPlot bootscan graphs with reference sequences. The recombination break-point is between the strain of Kato and TA716- like, at the nucleotide position 373 and 655 of the *Ot* TSA variable domain I-III.
